# Murepavadin induces envelope stress response and enhances the killing efficacies of β-lactam antibiotics by impairing the outer membrane integrity of *Pseudomonas aeruginosa*

**DOI:** 10.1128/spectrum.01257-23

**Published:** 2023-09-05

**Authors:** Xiaoya Wei, Jiacong Gao, Congjuan Xu, Xiaolei Pan, Yongxin Jin, Fang Bai, Zhihui Cheng, Iain L. Lamont, Daniel Pletzer, Weihui Wu

**Affiliations:** 1 State Key Laboratory of Medicinal Chemical Biology, Key Laboratory of Molecular Microbiology and Technology of the Ministry of Education, Department of Microbiology, College of Life Sciences, Nankai University, Tianjin, China; 2 Department of Biochemistry, University of Otago, Dunedin, New Zealand; 3 Department of Microbiology and Immunology, University of Otago, Dunedin, New Zealand; Yangzhou University, Yangzhou, Jiangsu, China

**Keywords:** *Pseudomonas aeruginosa*, murepavadin, β-lactam antibiotics, antibiotic resistance

## Abstract

*Pseudomonas aeruginosa* is a ubiquitous opportunistic pathogen that can cause a variety of acute and chronic infections. The bacterium is highly resistant to numerous antibiotics. Murepavadin is a peptidomimetic antibiotic that blocks the function of *P. aeruginosa* lipopolysaccharide (LPS) transport protein D (LptD), thus inhibiting the insertion of LPS into the outer membrane. In this study, we demonstrated that sublethal concentrations of murepavadin enhance the bacterial outer membrane permeability. Proteomic analyses revealed the alteration of protein composition in bacterial inner and outer membranes following murepavadin treatment. The antisigma factor MucA was upregulated by murepavadin. In addition, the expression of the sigma E factor gene *algU* and the alginate synthesis gene *algD* was induced by murepavadin. Deletion of the *algU* gene reduces bacterial survival following murepavadin treatment, indicating a role of the envelope stress response in bacterial tolerance. We further demonstrated that murepavadin enhances the bactericidal activities of β-lactam antibiotics by promoting drug influx across the outer membrane. In a mouse model of acute pneumonia, the murepavadin–ceftazidime/avibactam combination showed synergistic therapeutic effect against *P. aeruginosa* infection. In addition, the combination of murepavadin with ceftazidime/avibactam slowed down the resistance development. In conclusion, our results reveal the response mechanism of *P. aeruginosa* to murepavadin and provide a promising antibiotic combination for the treatment of *P. aeruginosa* infections.

**Importance:**

The ever increasing resistance of bacteria to antibiotics poses a serious threat to global public health. Novel antibiotics and treatment strategies are urgently needed. Murepavadin is a novel antibiotic that blocks the assembly of lipopolysaccharide (LPS) into the *Pseudomonas aeruginosa* outer membrane by inhibiting LPS transport protein D (LptD). Here, we demonstrated that murepavadin impairs bacterial outer membrane integrity, which induces the envelope stress response. We further found that the impaired outer membrane integrity increases the influx of β-lactam antibiotics, resulting in enhanced bactericidal effects. In addition, the combination of murepavadin and a β-lactam/β-lactamase inhibitor mixture (ceftazidime/avibactam) slowed down the resistance development of *P. aeruginosa*. Overall, this study demonstrates the bacterial response to murepavadin and provides a new combination strategy for effective treatment.

## INTRODUCTION

Infection of *Pseudomonas aeruginosa* is a major cause of morbidity and mortality in individuals with cystic fibrosis (CF), compromised immunity, healthcare-associated pneumonia, and chronic obstructive pulmonary disease (COPD) (1 – 3). The bacterium is intrinsically resistant to a variety of antibiotics, with resistance attributed to low membrane permeability, multi-drug efflux systems, and chromosomally encoded antibiotic modification/degradation enzymes. Mutations and horizontal acquisition of antibiotic resistance genes further enhance resistance (4). In 2017, carbapenem-resistant *P. aeruginosa* was listed by the World Health Organization (WHO) as one of the most critical pathogens for which new antibiotics are urgently needed (5, 6).

Murepavadin (POL7080) is a novel cyclic β hairpin peptide composed of 14 amino acids (7). It is currently developed for inhalation therapy in CF patients (https://spexisbio.com/pol7080/). Murepavadin specifically acts on the *P. aeruginosa* outer membrane protein LptD, which is a transporter of lipopolysaccharide (LPS), leading to defective assembly of LPS and ultimately cell death (7, 8). *P. aeruginosa* treated with sublethal concentrations of murepavadin has shown to accumulate LPS in the cytoplasmic membrane (9). The cyclic peptide has been shown to be effective against extensively drug-resistant (XDR) *P. aeruginosa*, including carbapenemase producers and colistin-resistant strains (10). However, *in vitro* passaging assays demonstrated rapid development of resistance to murepavadin. Mutations in the *pmrB* gene confer high levels of resistance to murepavadin and also colistin (11). PmrB is an integral membrane sensor kinase, forming a two-component regulatory system with PmrA, which is one of the major regulators of lipid A modifications in Gram-negative bacteria (12). In response to low Mg^2+^ conditions and cationic antimicrobial peptides, PmrB undergoes conformational change in its HAMP (histidine kinase, adenylyl cyclase, methyl-accepting chemotaxis protein, and phosphatase) domain, leading to autophosphorylation. The phosphate group is then transferred to the cognate response regulator PmrA. The phosphorylated PmrA directly upregulates the *arnBCADTEF-ugd* operon, which subsequently adds 4-amino-4-deoxy-L-arabinose (Ara4N) to the core and lipid A regions, reducing the negative charge of LPS and thus the affinity to cationic molecules (13 – 17). Mutations in *pmrB* can result in the protein becoming constitutively active, leading to the addition of Ara4N to LPS even in the absence of low Mg^2+^ conditions.

Combination therapies may improve treatment outcomes due to synergy and suppress resistance development (18). Synergy can result from enhanced bindings of antibiotics with their targets (19). In addition, antibiotics targeting the same cellular process can lead to synergy. Sulfamethoxazole and trimethoprim inhibit dihydropteroate synthase and dihydrofolate reductase, respectively, both of which are required for folate synthesis. Simultaneous blocking of the two enzymes results in synergy (20). Antibiotics that target different cellular processes may also have synergistic effects (19). Synergy has been found between β-lactam and aminoglycoside antibiotics. Inhibition of peptidoglycan synthesis by β-lactams facilitates the uptake of aminoglycosides that subsequently inhibit protein translation (21). Colistin promotes the uptake of rifamycin, glycopeptide, and macrolide antibiotics by increasing the outer membrane permeability, leading to synergy (22).

Here, we explored the effects of murepavadin on bacterial membrane integrity and protein composition. We demonstrated that the peptide enhances the uptake of β-lactam antibiotics, leading to synergy. In addition, we found that the combination of murepavadin and ceftazidime-avibactam slows down the resistance development of *P. aeruginosa*. Overall, our results provide a strategy to improve the therapeutic efficacy of murepavadin while slowing down the development of antibiotic resistance.

## RESULTS AND DISCUSSION

### Murepavadin increases the outer membrane permeability in *P. aeruginosa*

**Fig 1:**
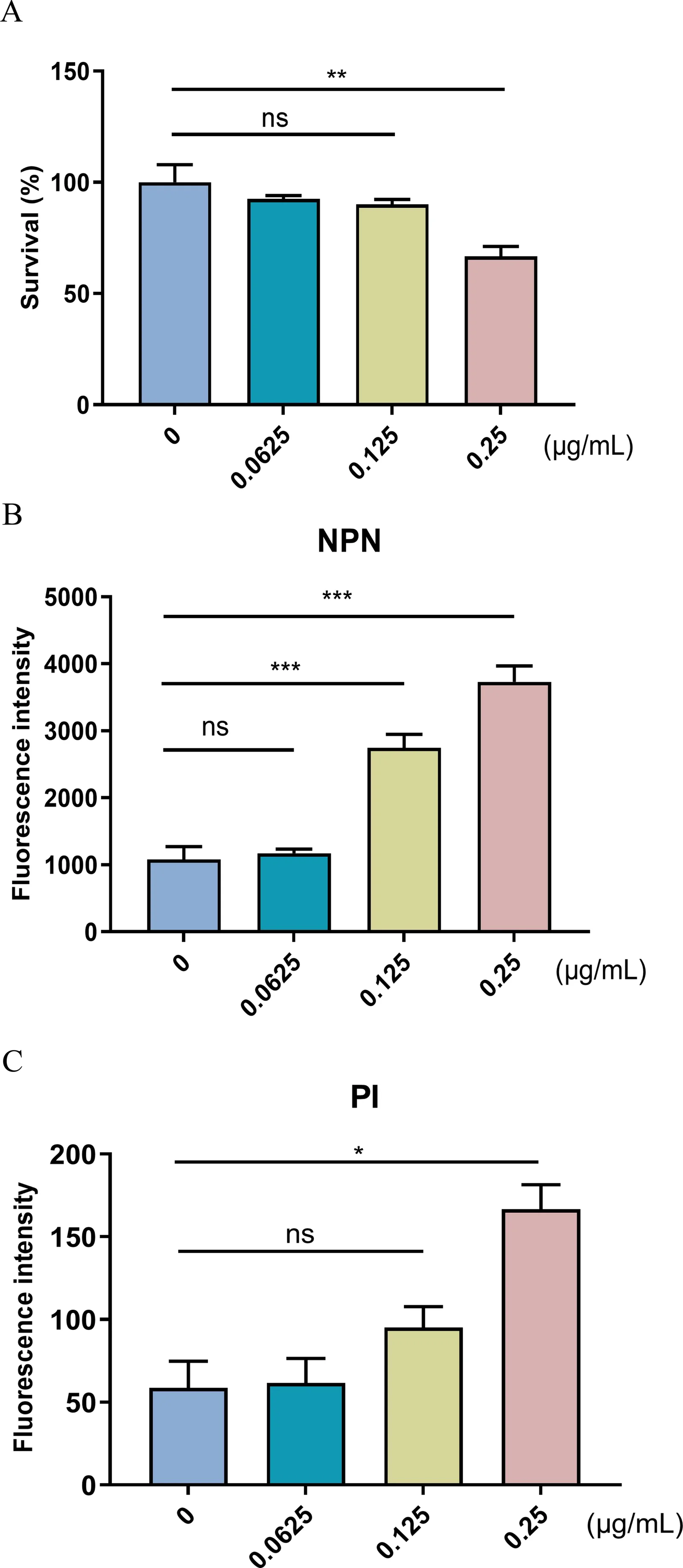
Murepavadin increases the permeability of the outer membrane. (**A**) Survival of *P. aeruginosa* PA14 following murepavadin treatment. NPN (**B**) and PI (**C**) staining following murepavadin treatment. ns, not significant; *, *P* < 0.05; **, *P* < 0.01; ***, *P* < 0.001, by Student’s *t*-test.

Since murepavadin inhibits LPS transport and subsequent insertion into the outer membrane (7, 8), we assessed its effect on bacterial membrane integrity. The MIC of murepavadin for the wild-type reference strain PA14 is 0.0625 µg/mL. Treatment with murepavadin at concentrations of 0.0625, 0.125, and 0.25 µg/mL (corresponding to 1×, 2×, and 4× MIC) for 1 hr resulted in 92.53%, 90.05%, and 66.7% survival, respectively (Fig. 1A). At 0.125 and 0.25 µg/mL, murepavadin increased N-phenyl-1-naphthylamine (NPN) staining (Fig. 1B), indicating increased outer membrane permeability. Propidium iodide (PI) staining demonstrated that the inner membrane permeability was increased by treatment with murepavadin at 0.25 µg/mL, which might relate to the reduced survival (Fig. 1A and C). Collectively, these results demonstrate that murepavadin mainly impairs the outer membrane integrity.

### Murepavadin induces AlgU-mediated envelope stress response in *P. aeruginosa*

**Fig 2:**
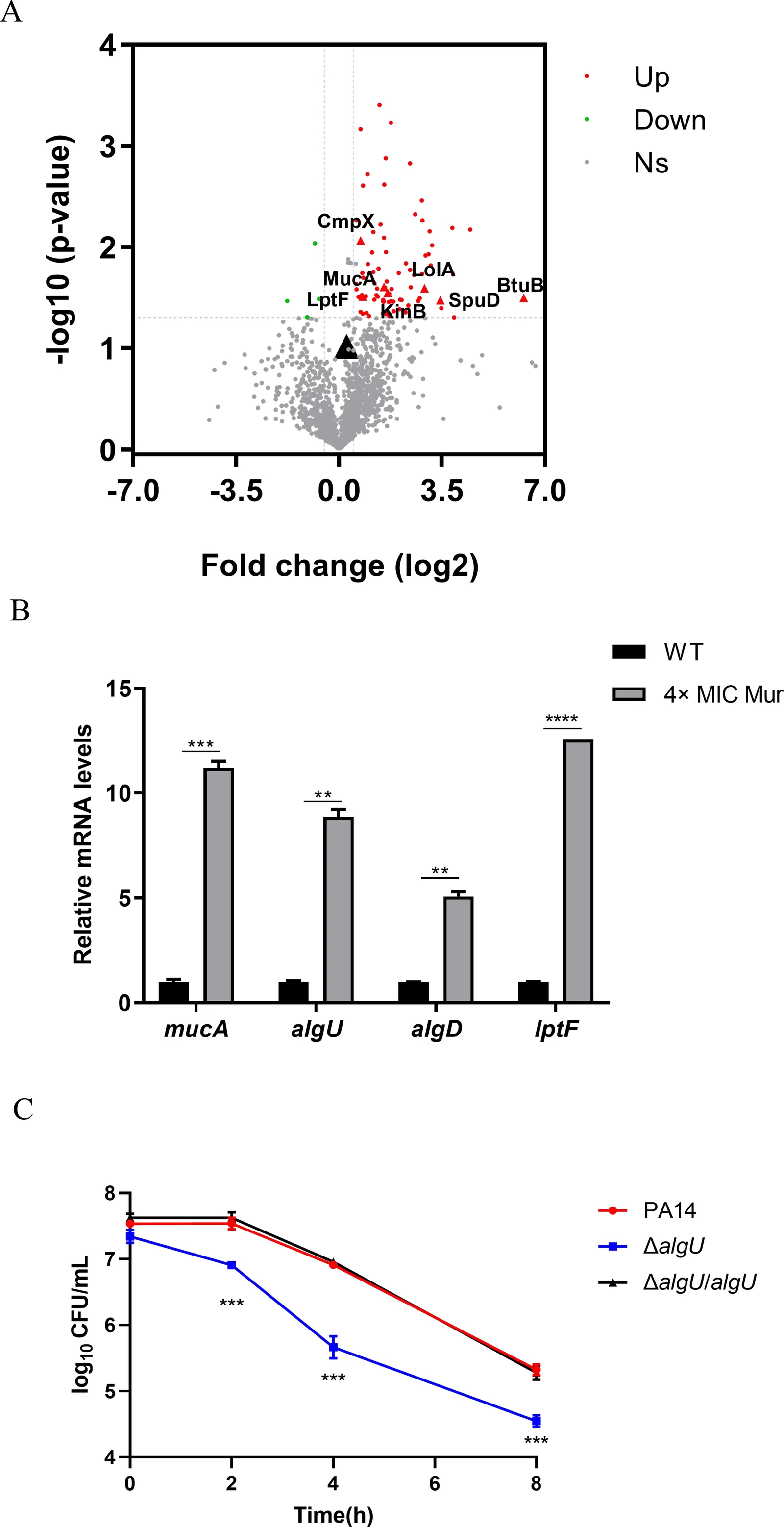
Murepavadin induces the AlgU pathway. (**A**) Volcano plot depicting membrane proteins variation with or without murepavadin treatment. The x-axis shows log_2_ changes of proteins in outer membrane, periplasm, and inner membrane after murepavadin treatment. Red and green dots indicate significantly (*P* < 0.05) upregulated and downregulated proteins. (**B**) The mRNA levels of genes in the AlgU regulatory pathway were determined by qRT-PCR. Data represent the mean ± standard deviation of results from three samples. **, *P* < 0.01; ***, *P* < 0.001; ****, *P* < 0.0001, by Student’s *t*-test. WT, wild-type PA14; Mur, murepavadin. (**C**) Time-kill curves of murepavadin against indicated strains. Bacteria were treated with 0.5 µg/mL murepavadin, and bacterial numbers were determined by plating at indicated time points. ***, *P* < 0.001, compared to wild-type PA14 and the complemented strain at the corresponding time points by Student’s *t*-test.

A previous study demonstrated that the inhibition of LPS transport by murepavadin results in the accumulation of LPS in the inner membrane (9), which might affect protein composition in both outer and inner membranes. To understand the bacterial response, we performed proteomic analysis on the membrane proteins before and after treatment with a sublethal dose of murepavadin. Wild-type PA14 was treated with 0.25 µg/mL murepavadin for 1 hr before protein isolation. After the treatment, more than 66% of cells were able to form colonies (Fig. 1A). Thus, this condition might impose a strong stress on the bacteria while the proteins being measured were mainly from live bacteria. Among the proteins localized in the outer and inner membranes as well as periplasm, 26 proteins were upregulated and two proteins were downregulated (fold change > 1.5), respectively (Fig. 2A; Table S1) (23). Notably, the amount of the antisigma factor MucA that inhibits the extra-cytoplasmic sigma factor AlgU was increased by approximately threefold after murepavadin treatment. The *mucA* and *algU* genes are in the *algU-mucA-mucB-mucC-mucD* operon, which is positively regulated by AlgU (24). In addition, the murepavadin-induced outer membrane protein gene *lptF* (PA14_16630) is directly activated by AlgU (25). Since the AlgU regulon plays an important role in bacterial envelope stress response, we focused our study on AlgU and its regulated genes (26, 27). Results from qRT-PCR verified that treatment with murepavadin upregulated the expression of *mucA*, *algU*, *lptF,* and the alginate biosynthesis gene *algD* that is also regulated by AlgU (Fig. 2B), indicating the activation of the AlgU-mediated envelope stress response.

To test whether the impaired outer membrane integrity serves as an activation signal for the AlgU-mediated response, we incubated wild-type PA14 with colistin for 30 min. Treatment with 1 µg/mL colistin increased NPN staining without altering PI staining (Fig. S1A). The treatment also increased the expression of *algU*, *mucA*, *algD,* and *lptF* by 1.8–3-fold (Fig. S1B), indicating the activation of the AlgU pathway.

We then examined the role of AlgU in bacterial resistance to murepavadin by constructing an *algU* in frame deletion mutant in wild-type PA14. Mutation of *algU* did not affect the MIC of murepavadin but reduced the bacterial survival (Table S2; Fig. 2C). Mutation of *algD* did not affect the MIC or bacterial survival (Table S2; Fig. S2). These results suggest a role of AlgU in bacterial tolerance to murepavadin, which might be attributed to the AlgU-mediated envelope stress response but independent of alginate production (28, 29).

### Murepavadin enhances the bactericidal effects of β-lactam antibiotics against *P. aeruginosa*

**Fig 3:**
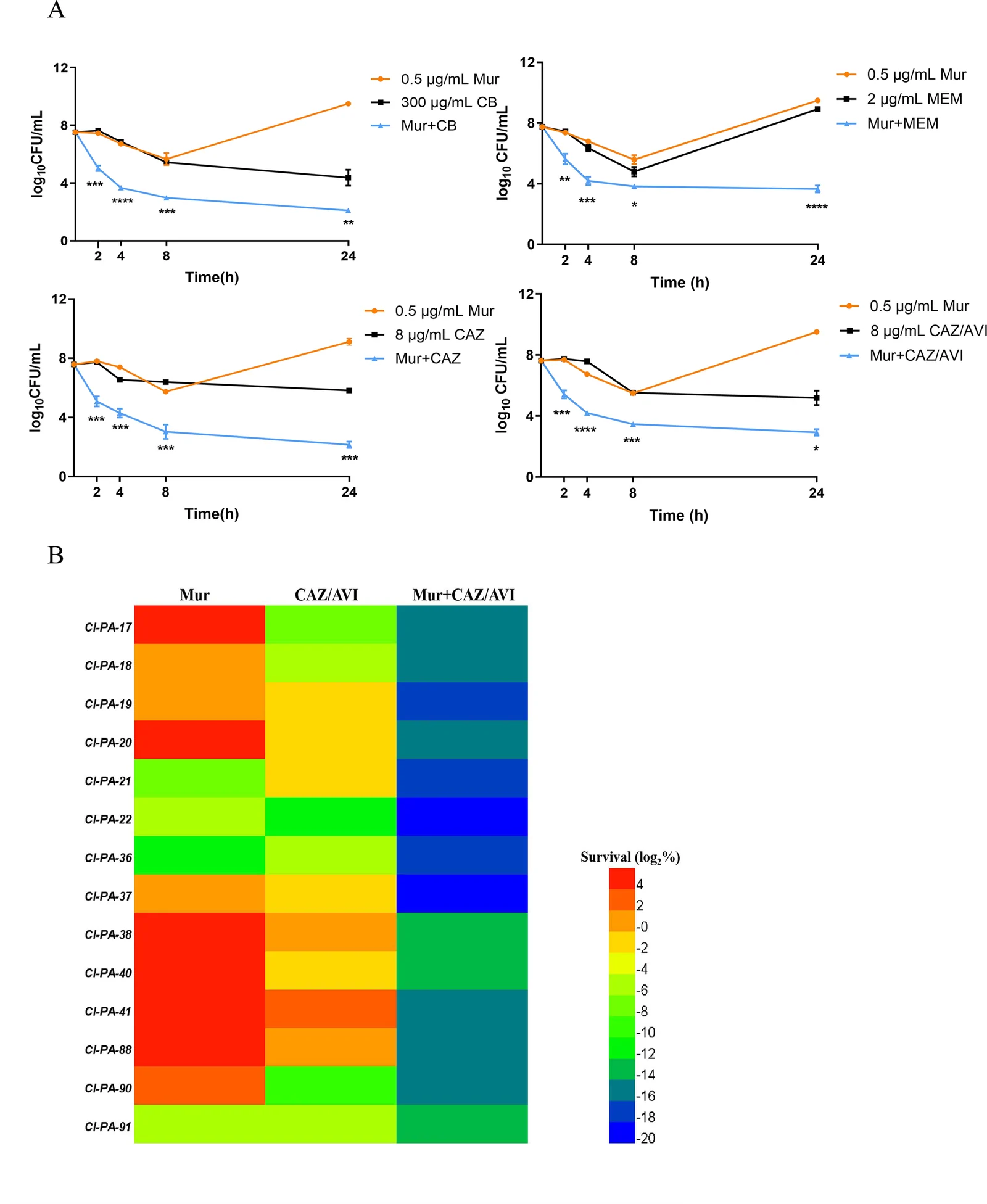
Murepavadin increases the bactericidal activity of β-lactam antibiotics. (**A**) Bactericidal activity of murepavadin in combination with indicated β-lactam antibiotics against PA14. *, *P* < 0.05; **, *P* < 0.01; ***, *P* < 0.001; ****, *P* < 0.0001 compared to the bacteria treated with the single antibiotic at the corresponding time points by Student’s *t*-test. (**B**) Bactericidal activities of murepavadin in combination with ceftazidime/avibactam against 14 carbapenem-resistant clinical strains. The bacteria were treated with or without murepavadin (0.5 µg/mL), ceftazidime (8 µg/mL)/avibactam (4 µg/mL), individually or in combination at 37°C. At 24 hr, bacterial samples were collected, and bacterial numbers were determined by plating. The colors indicate bacterial survival percentages. Mur, murepavadin; CB, carbenicillin; MEM, meropenem; CAZ, ceftazidime; AVI, avibactam.

Since murepavadin compromises outer membrane integrity (Fig. 1B and C), we hypothesized that it might enhance the efficacies of β-lactam antibiotics, which exert their bactericidal effects in the periplasm. Carbenicillin, meropenem, ceftazidime, and the β-lactam/β-lactamase inhibitor combination ceftazidime/avibactam were used at concentrations of 4× MIC, which are lower than their individual clinical breakpoints (30). Murepavadin was used at 0.5 µg/mL, which has been shown to inhibit the growth of 99.1% of the tested *P. aeruginosa* isolates (31). Murepavadin enhanced the bactericidal effects of the β-lactam antibiotics and ceftazidime/avibactam (Fig. 3A; Table S3). It has been demonstrated that ceftazidime/avibactam is effective against 73% carbapenem-resistant *P. aeruginosa* clinical isolates, which might be attributed to the inhibitory activity of avibactam against Ambler class A (including *Klebsiella pneumoniae* carbapenemases), class C, and some class D β-lactamases (32, 33). We then evaluated the killing efficacies of the murepavadin-ceftazidime/avibactam combination against 14 carbapenem-resistant clinical isolates, which carry *Klebsiella pneumoniae* carbapenemase (Table S4). Compared to murepavadin and ceftazidime/avibactam alone, the combination increased killing efficacies by 43 to 2.04 × 10^6^ fold and 74 to 3.08 × 10^5^ fold, respectively (Fig. 3B; Table S4 and S5).

**Fig 4:**
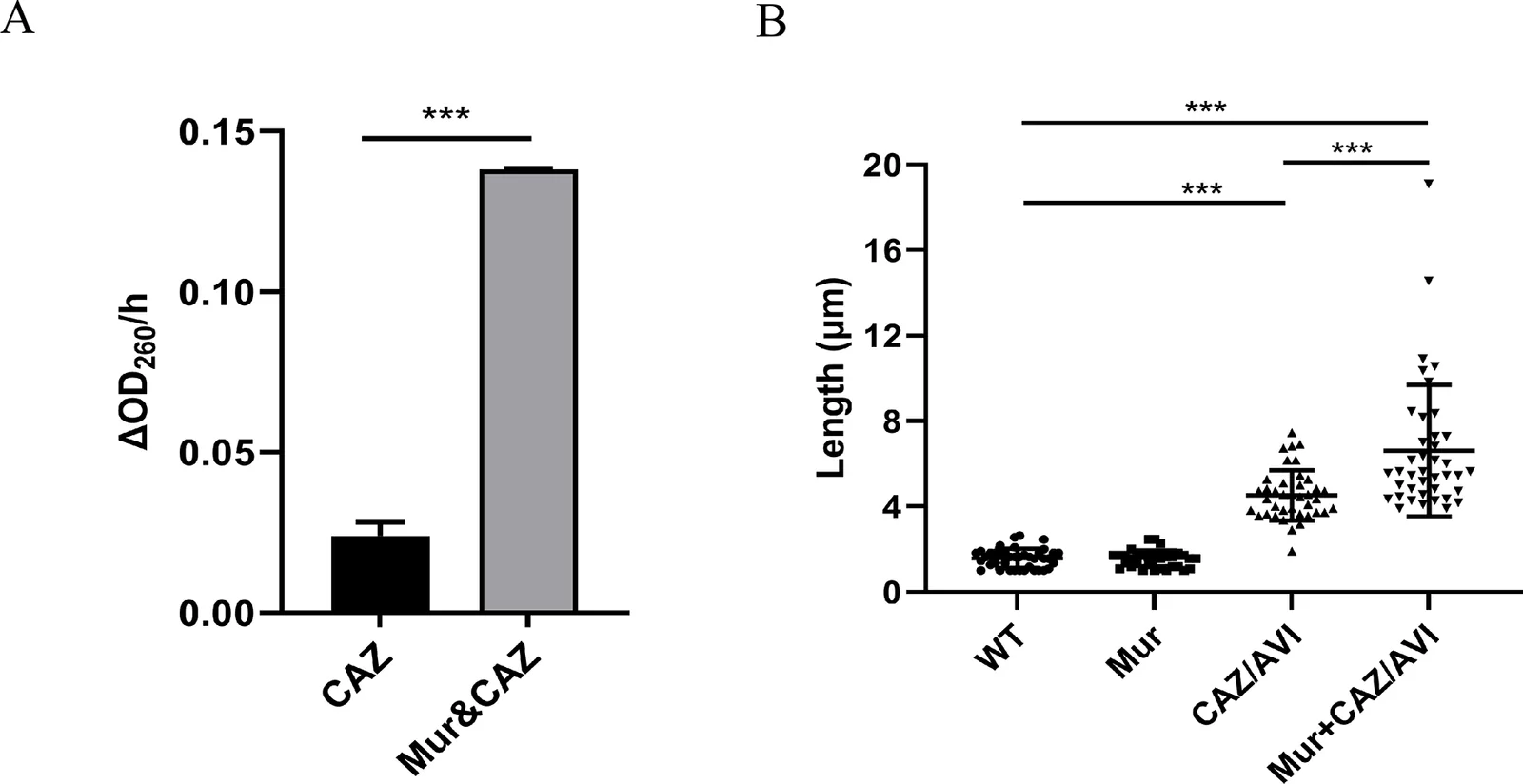
Murepavadin increases the influx of ceftazidime. (**A**) Hydrolysis rates of ceftazidime by *ampC* overexpressing *P. aeruginosa* (PA14/pUCP24-*ampC*) in the absence or presence of murepavadin. Data represent the mean ± standard deviation of three sample results. ***, *P* < 0.001 by Student’s *t*-test. (**B**) Statistical analysis of the length of PA14 cells following treatment with murepavadin, ceftazidime/avibactam, alone or in combination. ***, *P* < 0.001, by Student’s *t*-test. Mur, murepavadin; CAZ, ceftazidime; AVI, avibactam.

We then determined the influx rates of ceftazidime by using a β-lactamase (*ampC*) overexpressing PA14 as previously described (34, 35). The hydrolysis of ceftazidime by live bacteria corresponds to influx of the drug (34, 35). The presence of murepavadin enhanced the hydrolysis of ceftazidime by 5.7-fold (Fig. 4A). Meanwhile, the hydrolysis of ceftazidime in the bacterial supernatant was negligible (Fig. S3), indicating that murepavadin treatment did not cause extracellular leakage of the AmpC β-lactamase. Moreover, it has been demonstrated that ceftazidime causes filamentation of *P. aeruginosa* (36). This prompted us to further investigate the cell morphology. Murepavadin increased the bacterial length following ceftazidime/avibactam treatment (Fig. 4B; Fig. S4). In combination, these results suggest that murepavadin enhances the bactericidal effects of β-lactam antibiotics by promoting drug influx.

Among the murepavadin-induced outer membrane proteins, PA14_47800 was the most upregulated protein (Fig. 2A; Table S1). PA14_47800 is homologous to the *E. coli* TonB-dependent vitamin B12 transporter BtuB (37, 38). However, overexpression of *PA14_47800* (*btuB*) did not increase the bacterial survival following murepavadin treatment (Fig. S5). Another murepavadin-induced protein LptF (PA14_16630) is an OmpA-like outer membrane protein (25, 37). Mutations in OmpA contribute to cefiderocol resistance in *Klebsiella pneumoniae* (39). We suspected that LptF might play a role in the enhanced susceptibility to β-lactam antibiotics. Western blot results verified the increased expression and membrane abundance of LptF following murepavadin treatment (Fig. S6A). Overexpression of *lptF* in wild-type PA14 reduced the bacterial survival by approximately 5-fold after treatment with ceftazidime/avibactam for 8 hr (Fig. S6B). In contrast, overexpression of *lptF* increased the bacterial survival by approximately 10-fold 8 hr after murepavadin treatment (Fig. S6C). These results demonstrated a role of LptF in the altered susceptibilities to ceftazidime/avibactam and murepavadin.

### The murepavadin-ceftazidime/avibactam combination displays a synergistic effect against PA14 *in vivo*

**Fig 5:**
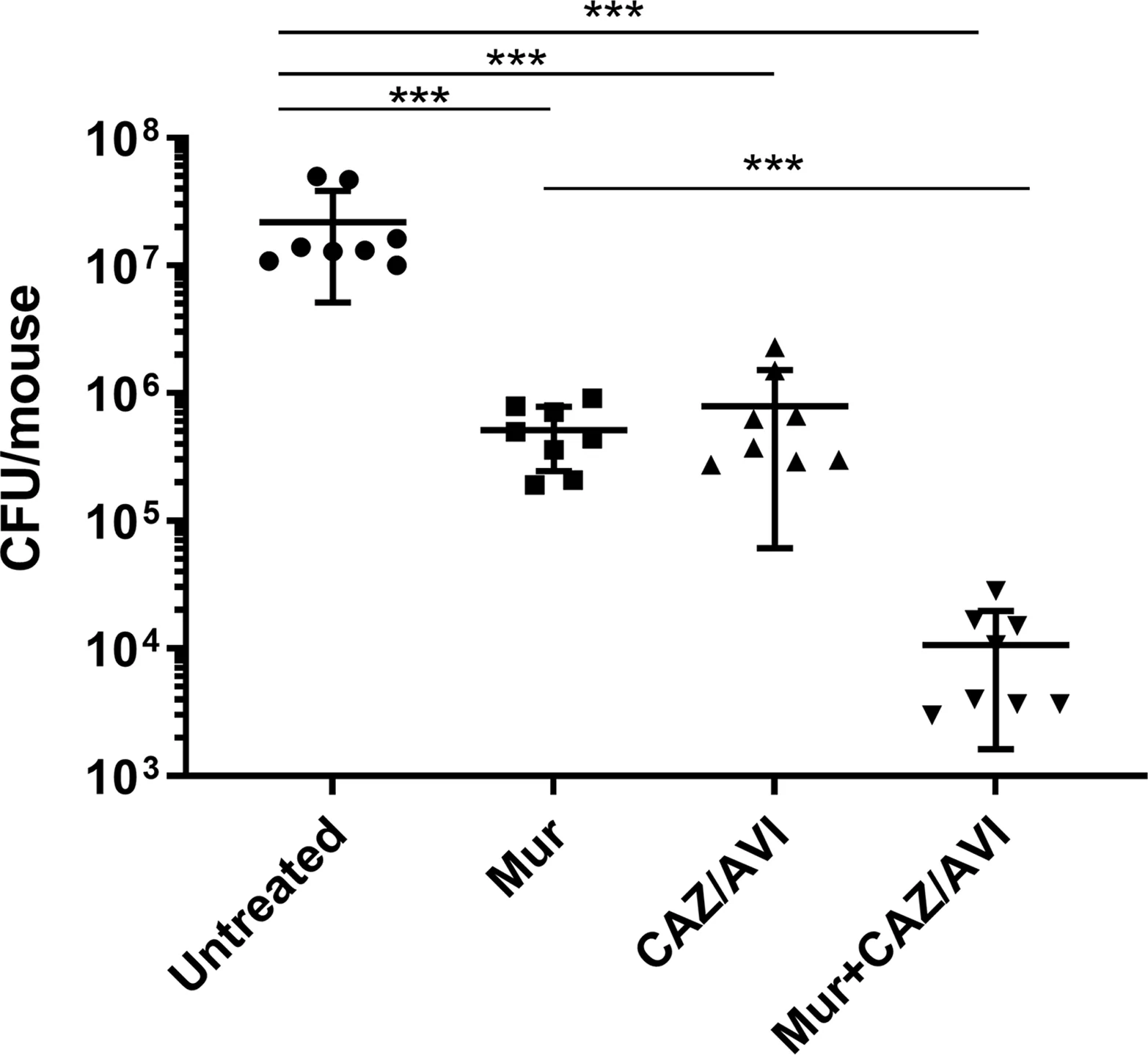
The murepavadin-ceftazidime/avibactam combination promotes killing of *P. aeruginosa in vivo*. Bacterial loads of PA14 in the lungs of mice 13 hr after treatment with murepavadin (0.25 mg kg^−1^), ceftazidime/avibactam (7.5 mg kg^−1^/1.875 mg kg^−1^), alone or in combination (*n* = 8 per group). The average CFU of the bacteria is presented as horizontal lines. ***, *P* < 0.001 by Student’s *t*-test. Mur, murepavadin; CAZ, ceftazidime; AVI, avibactam.

We next evaluated the *in vivo* treatment efficacy of the drug combination against *P. aeruginosa* infection in a murine acute pneumonia model (40, 41). The dose of murepavadin (0.25 mg/kg) was used as previously described in a mouse infection model (9). For ceftazidime-avibactam, the recommended single dose for adult humans is 2 g ceftazidime plus 0.5 g avibactam through the intravenous route (42, 43). Based on the Meeh-Rubner equation (44), the equivalent doses for intravenous injection of mice are 225 mg/kg ceftazidime and 56 mg/kg avibactam. In our experiments, ceftazidime-avibactam and murepavadin were administered intranasally, and so, we tested lower doses. Doses of 7.5 mg/kg ceftazidime with 1.875 mg/kg avibactam resulted in similar CFU reduction to murepavadin (Fig. 5). Each mouse was infected with 4 × 10^6^ CFU of wild-type PA14 intranasally; 3 hr after infection, murepavadin and ceftazidime/avibactam were administered intranasally, alone or in combination. Treatment with murepavadin and ceftazidime/avibactam alone reduced the mean bacterial loads by 42-fold and 28-fold, respectively, whereas the combined treatment reduced the mean bacterial load by 2047-fold (Fig. 5), demonstrating a synergistic bactericidal effect.

### The murepavadin-ceftazidime/avibactam combination slows down the resistance development

**Fig 6:**
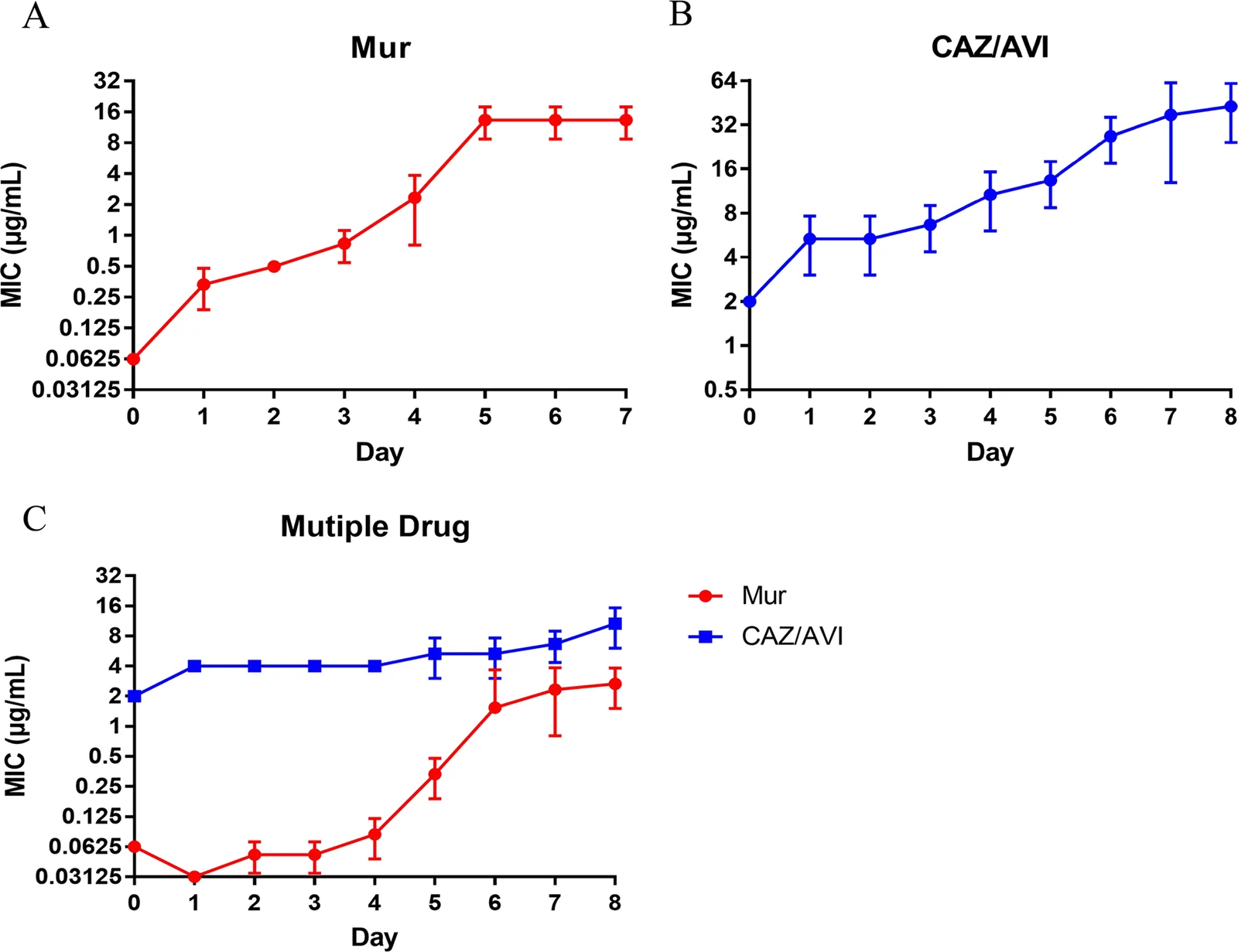
Effects of murepavadin in combination with ceftazidime/avibactam on the development of resistance in PA14. Passaging of PA14 in murepavadin (Mur) (**A**), ceftazidime/avibactam (CAZ/AVI) alone or the combination (Mur:CAZ/AVI = 1:10). MICs of the corresponding individual antibiotics were measured daily. Data represent the mean ± standard deviation of results from three repeats. Error bars indicate SEM. Mur, murepavadin; CAZ, ceftazidime; AVI, avibactam.

Antibiotic combinations with synergistic effects might suppress resistance development (18). We thus evaluated the resistance development of PA14 by *in vitro* passaging assays. In the presence of murepavadin alone, the MIC was increased from 0.0625 µg/mL to 12 ± 4 µg/mL within 5 d and remained stable afterwards (Fig. 6A). For ceftazidime/avibactam, the MIC for PA14 was increased from 2 µg/mL to 48 ± 16 µg/mL within 8 d (Fig. 6B). However, combination of the two antibiotics resulted in the MICs of murepavadin and ceftazidime/avibactam at 3 ± 1 µg/mL and 12 ± 4 µg/mL after 8 d, respectively, indicating a suppression of resistance development (Fig. 6C).

### Conclusions

Murepavadin is a cyclic peptide antibiotic that disrupts the lipid asymmetry of the outer membrane bilayer by selectively inhibiting the *P. aeruginosa* LPS transport machinery component LptD (7). Here, we found that treatment with murepavadin increases the outer membrane permeability, which might be due to inhibition of LPS insertion into the outer membrane and alteration of the phospholipid and protein components in the outer membrane. Mislocalized LPS activates the AlgU pathway (45), and both murepavadin and colistin activated the AlgU-mediated envelope stress response. Therefore, murepavadin-inhibited LPS transportation and the subsequent impaired outer membrane integrity likely play a major role in activating the AlgU-mediated envelope stress response. We further demonstrated that AlgU contributes to bacterial survival in the presence of murepavadin, likely attributed to the role of the envelope stress response in maintaining membrane integrity (45). Murepavadin enhances the bactericidal activities of β-lactam antibiotics by promoting drug influx. The AlgU-regulated OmpA family protein gene *lptF* is upregulated by murepavadin. Overexpression of *lptF* in wild-type PA14 increases bacterial survival following murepavadin treatment but reduces survival following ceftazidime/avibactam treatment. We suspect that the increased abundance of LptF in the outer membrane might contribute to the maintenance of outer membrane integrity while increasing the influx of β-lactam antibiotics. Further studies are warranted to examine whether LptF directly facilitates the diffusion of β-lactam antibiotics. Previous studies demonstrated that AlgU regulates more than 500 genes (46, 47). Besides alginate biosynthesis genes, AlgU regulates two-component regulatory systems, FimS-AlgR and KinB-AlgB (48). In addition, AlgU regulates genes involved in LPS biosynthesis (*wbpH*, *wbpD*, *wzz*, and *rmlD*) and peptidoglycan biosynthesis (*mrcB*, *mpl,* and *mdoH*) (28), which might contribute to the bacterial response to murepavadin. Further studies are needed to elucidate the roles of those genes in resistance.

Murepavadin enhances the bactericidal effect of ceftazidime/avibactam against multiple carbapenem-resistant clinical isolates. Combination of murepavadin and ceftazidime/avibactam displays synergistic therapeutic effects in a murine acute pneumonia model and slows down the resistance development *in vitro*. Overall, our results reveal the bacterial response to murepavadin and a combination therapeutic strategy with synergy and the ability to slow down the resistance development.

## MATERIALS AND METHODS

### Bacteria strains and growth conditions

The bacteria strains, primers, and plasmids used in this study are listed in Table S5. *P. aeruginosa* strains were grown in lysogeny broth (LB) or cation-adjusted Mueller-Hinton broth (CA-MHB) at 37°C with shaking at 200 rpm unless otherwise indicated. Detailed methods of gene deletion and complementation are provided in the supplementary material.

### Antimicrobial susceptibility test

The minimal inhibitory concentrations (MICs) of selected antibiotics were determined in triplicate using the standard serial 2-fold dilution method in CA-MHB (Cation adjusted-Mueller Hinton Broth) in accordance with Clinical and Laboratory Standards Institute (CLSI) guidelines (30). Murepavadin trifluoroacetic acid was purchased from MCE (MedChemExpress, China). Avibactam was purchased from Meilunbio (China), and other antibiotics were purchased from Macklin (China).

### Time-kill assays

Overnight cultures of bacteria were grown to exponential growth phase (OD_600_ = 1.0) in LB broth. The bacteria were adjusted to 10^8^ CFU/mL in a test tube containing 2 ml fresh CA-MHB and subjected to antibiotic treatment. The bacterial suspension was incubated at 37°C with shaking. At indicated time points (0, 2, 4, 8, and 24 hr), bacterial survivors were determined by plating. All experiments were performed in triplicate.

### Assessment of the outer membrane permeability

The integrity of the outer membrane was measured by the NPN absorption assay with minor modifications (49). Briefly, bacterial overnight cultures were diluted 1: 100 into CA-MHB and grown to an OD_600_ of 1 at 37°C. The bacteria were adjusted to an OD_600_ of 0.5 in fresh CA-MHB and incubated at 37°C for 1 hr with or without murepavadin at concentrations of 0.0625 µg/mL, 0.125 µg/mL, or 0.25 µg/mL. For colistin treatment, the bacteria at OD_600_ of 0.5 were incubated at 37°C for 0.5 hr in CA-MHB with or without colistin at concentrations of 0.25 µg/mL (1× MIC), 0.5 µg/mL, and 1 µg/mL. The cells were then washed three times with 5 mM GHEPES buffer (Caisson Labs) containing 5 mM glucose and resuspended in the same buffer. The fluorescent probe NPN (Macklin) was added to the cells at a final concentration of 10 µM. The fluorescence was measured using an excitation wavelength of 350 nm and an emission wavelength of 420 nm with a fluorometer (Varioskan Flash; Thermo Scientific). All the tests were performed in triplicate.

### Assessment of inner membrane permeability

The integrity of the inner membrane was measured by the PI staining assay as previously described with minor modifications (50). Briefly, the overnight cultured bacteria were diluted 1: 100 into CA-MHB and grown to logarithmic phase (OD_600_ = 1) at 37°C. The bacteria were adjusted to an OD_600_ = 0.5 in fresh CA-MHB and incubated at 37°C for 1 hr with or without murepavadin at concentrations of 0.0625 µg/mL, 0.125 µg/mL, or 0.25 µg/mL, or incubated with or without colistin at concentrations of 0.25 µg/mL, 0.5 µg/mL, or 1 µg/mL at 37°C for 0.5 hr. The cells were then washed three times and resuspended in phosphate-buffered saline (PBS). The fluorescent dye propidium iodide (PI) (PI, MCE) was added to the cells at a final concentration of 10 µM, followed by incubation at 25°C under static conditions for 30 min. Fluorescence was measured using an excitation wavelength of 535 nm and an emission wavelength of 615 nm with a fluorometer (Varioskan Flash; Thermo Scientific). All the tests were performed in triplicate.

### RNA isolation and quantitative real-time PCR (qRT-PCR)

Overnight cultured bacteria were diluted 1: 100 into CA-MHB and grown to logarithmic phase (OD_600_ = 1) at 37°C. The bacteria were adjusted to an OD_600_ of 0.5 in fresh CA-MHB and incubated at 37°C with 0.25 µg/mL murepavadin for 1 hr or with 1 µg/mL colistin for 0.5 hr. Subsequently, bacteria were harvested by centrifugation at 12,000 g for 2 min, and the total RNA was extracted with a Bacteria Total RNA Kit (Zoman, Biotec, Beijing, China); 1 µg total RNA was reverse transcribed to cDNA at 55°C using random primers and the PrimeScript Reverse Transcriptase (TaKaRa, Dalian, China). Specific primers (Table S6) were used for qRT-PCR with the cDNA and SYBR Premix Ex Taq II™ (TaKaRa). The *rpsL* gene that encodes the 30S ribosomal protein was used as the internal control. Results were measured and analyzed using the CFX Connect real-time system (Bio-Rad, USA).

### Proteomic analysis of bacterial membrane proteins and western blotting

Overnight bacterial cultures were diluted 1: 100 into CA-MHB and incubated to the logarithmic phase (OD_600_ = 1) at 37°C. Bacteria were adjusted to an OD_600_ of 0.5 in fresh CA-MHB and incubated at 37°C for 1 hr with or without 0.25 µg/mL murepavadin. Bacterial membrane proteins were extracted by the bacterial membrane protein extraction kit (BestBio, Shanghai, China), and the concentrations were determined with the BCA kit (Beyotime, Shanghai, China). Proteomic analyses of bacterial membrane proteins by the quantification of data-dependent acquisition protein were performed by BGI Genomics (Shenzhen, China).

Equivalent amounts of total proteins and membrane proteins from bacteria were mixed with loading buffer, boiled at 99°C for 10 min. The proteins were separated by 12% SDS-polyacrylamide gel electrophoresis (SDS-PAGE) followed by transferring onto a polyvinylidene difluoride (PVDF) membrane (Millipore, USA). The membrane was incubated with a mouse monoclonal anti-His antibody (Millipore, USA) or a mouse monoclonal anti-RNA polymerase α antibody (Biolegend, USA) at room temperature for 1 hr. After washing with PBST (PBS with 0.05% Tween 20) for four times, the membrane was incubated with the HRP-conjugated goat antimouse secondary antibody (Promega, USA) at room temperature for 1 hr. The membrane was washed with PBST for four times, and then, the signals were detected with an ECL Plus kit (Millipore) and imaged using a Bio-Rad molecular imager (ChemiDocXRS).

### Ceftazidime influx assay

Influx of ceftazidime was measured as previously described with minor modifications (34). *P. aeruginosa* strains overexpressing the *ampC* gene were grown to an OD_600_ of 0.6 in CA-MHB, followed by incubation with or without 0.125 µg/mL murepavadin at 37°C for 1 hr; 1 mL of the bacterial cells were subjected to centrifugation at 10,000× g for 1 min. The supernatant was collected, and the bacterial cells were washed once and resuspended in 1 mL of PBS. The cells and supernatants were incubated with 64 µg/mL of ceftazidime at 37°C for 0.5 hr. Influx of ceftazidime was measured by the decrease of OD_260_ with a Varioskan Flash reader (Thermo Scientific, Netherlands). The change of the OD_260_ in the bacterial supernatant was used to determine the extracellular leakage of AmpC.

### Bacterial morphology observation

Overnight bacterial cultures were diluted 100-fold into fresh CA-MHB supplemented with murepavadin (0.03125 µg/mL), ceftazidime (1 µg/mL)/avibactam (4 µg/mL), individually or in combination and incubated at 37°C for 2.5 hr. The bacterial morphology was observed with light microscopy. The CellSens Dimension (Olympus, Japan) software was used to measure the length of each individual bacterium. Data were collected from 40 individual cells in three random fields.

### Murine lung infection model

The murine acute pneumonia model was performed as previously described (40, 41). Wild-type PA14 was grown in LB at 37°C overnight and subcultured into fresh LB medium to an OD_600_ of 1.0. The bacterial cells were then washed with PBS and adjusted to 2 × 10^8^ CFU/mL in PBS. Female BALB/c mice (Vital River) aged between 6 and 8 wk were housed at 20–22°C. Each mouse was injected intraperitoneally with 90 µL 7.5% chloral hydrate for anesthesia. To establish the lung infection, 20 µL bacterial suspension was inoculated intranasally into each mouse, resulting in 4  ×  10^6^ CFU per mouse. At this inoculum, mice survive more than 16 hr without antibiotic treatment (41); 3 hr post-infection (hpi), mice were anaesthetized again and administered intranasally with 20 µL of PBS, or PBS containing murepavadin (0.25 mg/kg), cedtazidime-avibactam (7.5 mg/kg-1.875 mg/kg), or the combination of the two drugs; 13 hr later, the mice were sacrificed by CO_2_ asphyxiation. The lungs were removed and homogenized in 1% proteose peptone (Solarbio, Beijing, China). The bacterial loads were enumerated by plating.

### *In vitro* evolution of murepavadin-resistant strains

Wild-type PA14 was propagated in the presence or absence of murepavadin; 10 µL of the overnight bacterial culture was subcultured into 1 mL of fresh CA-MHB with increasing concentrations of murepavadin (0.5×, 1×, 2×, and 4× MIC) with three parallels at each concentration. After 24 hr of aerobic incubation at 37°C, cells that were allowed to grow to an OD_600_ of 2.0 with the highest concentration of antibiotic were inoculated into fresh CA-MHB containing increasing concentrations of murepavadin (e.g., 1×, 2×, 4×, and 8× MIC) for another round of passaging. The passaging was repeated for 8 d. The bacteria were streaked on LB plates each day to obtain single colonies for MIC measurement. Meanwhile, another repeat passage in CA-MHB with antibiotic was used as a control.

In order to examine the resistant development for the antibiotic combination, the MIC of the combination was determined by mixing murepavadin and ceftazidime at a ratio of 1:10 in the presence of 4 µg/mL avibactam. The ratio was determined based on the plasma concentrations of the drugs (51, 52); 10 µL of the overnight bacterial culture was subcultured into 1 mL of fresh CA-MHB with increasing concentrations of the combinations of murepavadin with ceftazidime-avibactam (0.5×, 1×, and 2× MIC); 24 hr later, bacteria that were allowed to grow to an OD_600_ of 2.0 with the highest concentrations of antibiotics were inoculated into fresh CA-MHB containing increasing concentrations of antibiotics for another round of passaging. The passage was repeated for 8 d, and another repeat passage in CA-MHB was used as a control.

## SUPPLEMENTAL MATERIAL

Supplemental material spectrum.01257-23-s0001.pdf Fig. S1 to S6, Tables S1 to S6, and additional experimental details.

ASM does not own the copyrights to Supplemental Material that may be linked to, or accessed through, an article. The authors have granted ASM a non-exclusive, world-wide license to publish the Supplemental Material files. Please contact the corresponding author directly for reuse.
