# Supplementary material for: Murepavadin induces envelope stress response and enhances the killing efficacies of β-lactam antibiotics by impairing the outer membrane integrity of Pseudomonas aeruginosa
Source: Microbiol Spectr. 2023 Sep 5;11(5):e01257-23. doi: 10.1128/spectrum.01257-23 (PMC10581190; doi:10.1128/spectrum.01257-23)
Supplement: Supplemental material — Fig. S1 to S6, Tables S1 to S6, and additional experimental details. [file spectrum.01257-23-s0001.pdf]

Figure S1.

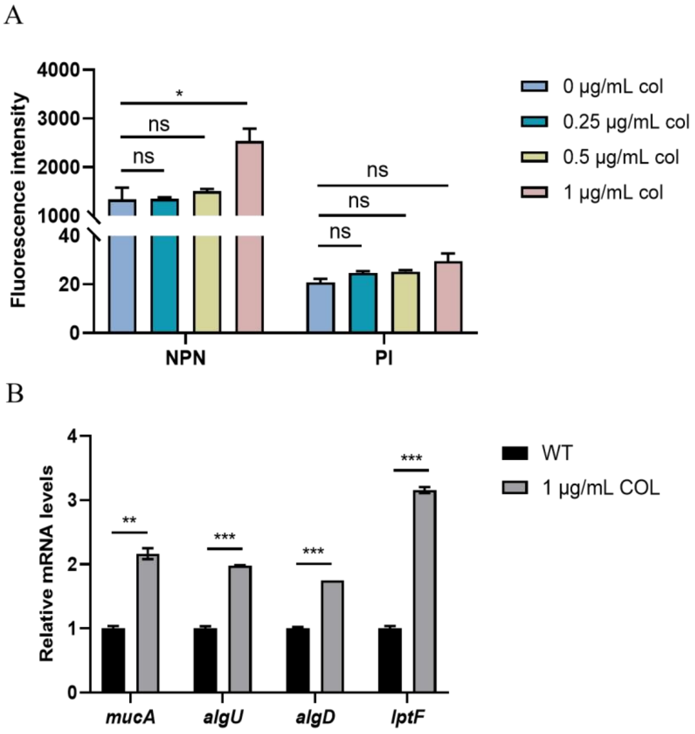

Fig. S1 Colistin increased the permeability of outer membrane and induced the AlgU pathway. (A) NPN and PI staining following colistin treatment. ns, not significant; *, P < 0.05 by Student's t-test. (B) The mRNA levels of genes in the AlgU regulatory pathway were determined by qRT-PCR. Data represent the mean ± standard deviation of results from three samples. **, P < 0.01; ***, P < 0.001 by Student's t-test. WT, wild type PA14; COL, colistin.

Figure S2.

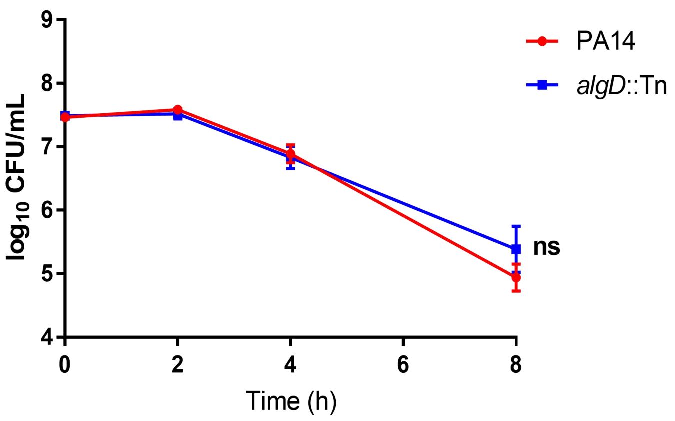

Fig. S2 Time-kill curves of murepavadin against PA14 and the *algD*::Tn mutatnt. The bacteria were treated with 0.5 μg/mL murepavadin. At 0, 2, 4, 8 h, bacterial samples were collected and bacterial survivors determined. ns, not significant by Student's t-test.

Figure S3.

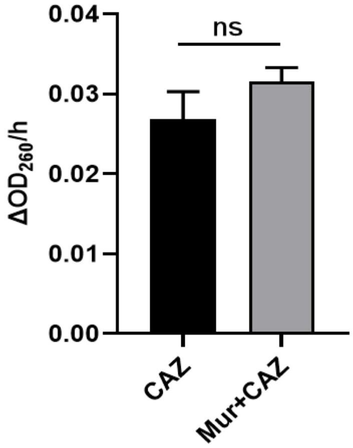

Fig. S3 Hydrolysis rates of ceftazidime in the supernatant of the *ampC* overexpressing PA14 in the absence or presence of murepavadin. Data represent the mean ± standard deviation of three sample results. ns, not significant by Student's t-test. CAZ, Ceftazidime; Mur, Murepavadin.

Figure S4.

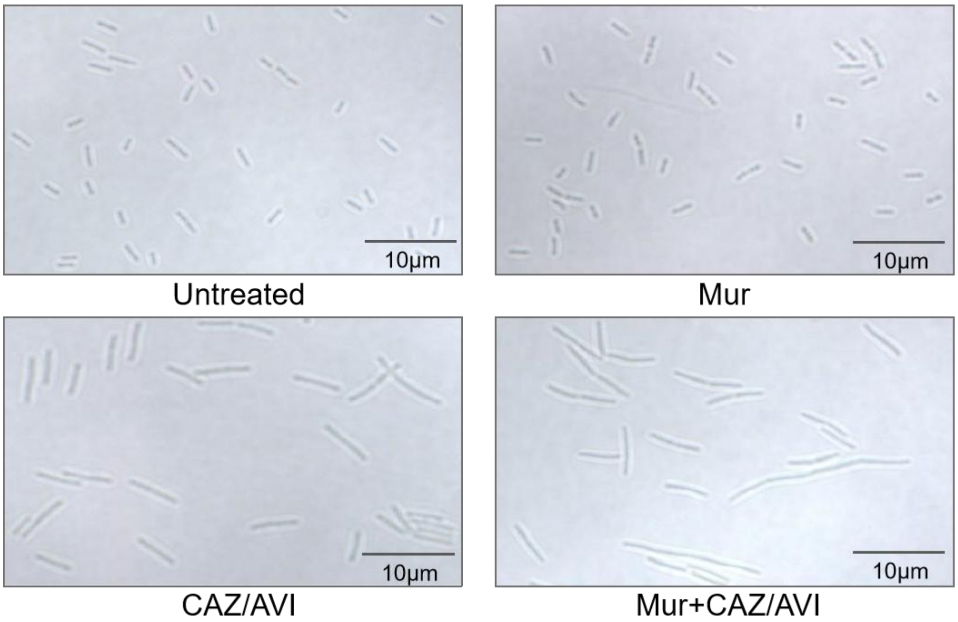

Fig. S4 Bacterial morphology analysis following treatment with murepavadin (0.03125 μg/mL), ceftazidime (1 μg/mL) /avibactam (4 μg/mL), alone or in combination for 2.5 hours. Mur, murepavadin; CAZ, ceftazidime; AVI, avibactam.

Figure S5.

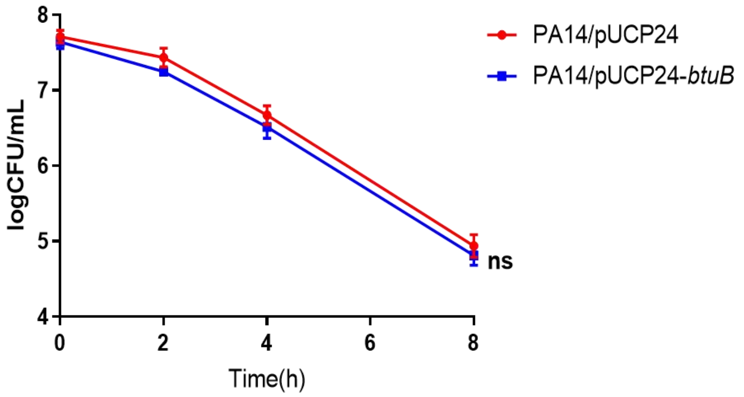

Fig. S5 Survival of PA14 containing the empty vector (PA14/pUCP24) or *btuB* overexpressing plasmid (PA14/pUCP24-*btuB*) following murepavadin treatment. The bacteria were treated with 0.5 μg/mL murepavadin. At 0, 2, 4, 8 h, bacterial samples were collected and the live bacteria numbers were determined by plating. ns, not significant by Student's t-test.

Figure S6.

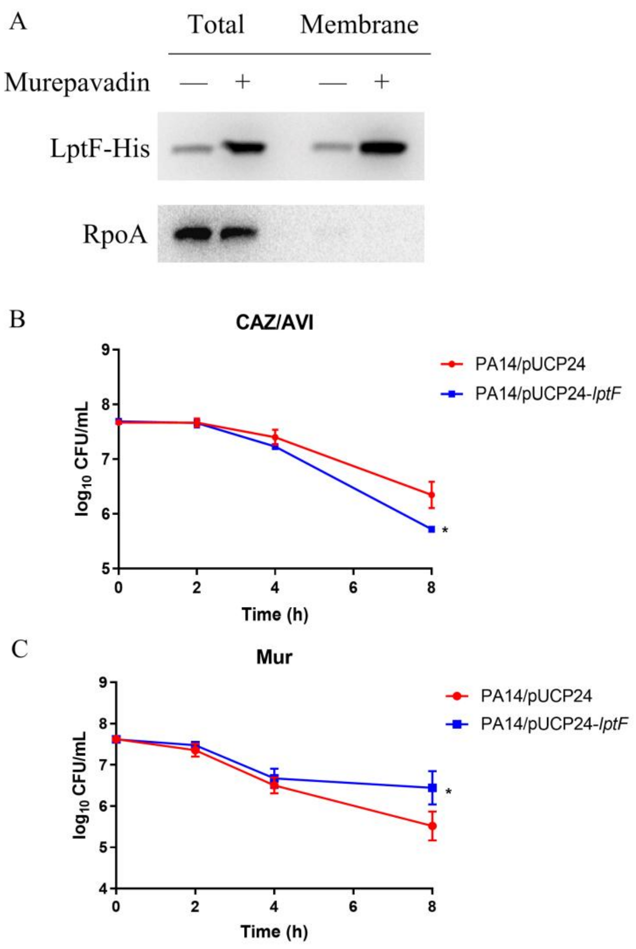

Fig. S6 Role of LptF in bacterial response to murepavadin. (A) The amount of total and membrane LptF after murepavadin treatment. (B) Bactericidal activity of ceftazidime/avibactam against indicated strains. The bacteria were treated with or without 4/4 μg/mL ceftazidime/avibactam for 0, 2, 4, 8 hours. The live bacteria numbers were determined by plating. (C) Bactericidal activities of murepavadin against indicated strains. The bacteria were treated with 0.5 μg/mL murepavadin for 0, 2, 4, 8 hours. The live bacteria numbers were determined by plating. Mur, murepavadin; CAZ, ceftazidime; AVI, avibactam. *, $P < 0.05$, by Student's t-test.

Table S1. Proteomic analysis: differentially expressed membrane proteins

Protein Name	Function Description	Fold Change (PA14-Mur/PA14)	P value
BtuB	Outer membrane cobalamin receptor protein	6.29	0.032
PA14_15120	Copper(I)-binding protein	3.91	0.050
YbeJ	ABC-type amino acid transport/signal transduction system, periplasmic component/domain	3.86	0.006
PA14_62690	Osmotically-inducible protein OsmY, contains BON domain	3.48	0.040
SpuD	Spermidine/putrescine-binding periplasmic protein	3.45	0.034
PA14_05510	Polyisoprenoid-binding periplasmic protein YceI	3.09	0.007
Eco	Serine protease inhibitor ecotin	3.05	0.012
LolA	Outer membrane lipoprotein-sorting protein	2.91	0.026
BraC	ABC-type branched-chain amino acid transport system, periplasmic component	2.85	0.005
DppA3	ABC-type transport system, periplasmic component	2.83	0.018
DsbA	Protein-disulfide isomerase	2.75	0.032
JF1	Phage-related protein, tail component	2.08	0.033
PA14_26780		1.80	0.026
LptF	Putative outer membrane protein	1.75	0.030
KinB	Signal transduction histidine kinase	1.67	0.028
PA14_09550	6-phosphogluconolactonase, cycloisomerase 2 family	1.59	0.001
MucA	Negative regulator of sigma E activity	1.54	0.025
PotA	ABC-type Fe3+/spermidine/putrescine transport systems, ATPase components	1.52	0.035
PA14_41130	ABC-type oligopeptide transport system, periplasmic component	1.51	0.035
AotP	ABC-type histidine transport system, ATPase component	1.33	0.031
PA14_28820	Uncharacterized conserved protein, tellurite resistance protein B (TerB) family	1.13	0.011

NarK2	Nitrate/nitrite transporter NarK	0.98	0.002
YedI	Uncharacterized membrane protein MutK, may be involved in DNA repair	0.83	0.020
TatC	Sec-independent protein secretion pathway component TatC	0.81	0.045
CmpX	Small-conductance mechanosensitive channel	0.74	0.009
PA14_52130	LPS O-antigen chain length determinant protein, WzzB/FepE family	0.67	0.031
PA14_69320	Membrane protein TerC, possibly involved in tellurium resistance	0.59	0.023
PA14_28050	Signal transduction histidine kinase, nitrate/nitrite-specific	-0.67	0.033
HelX	Thiol-disulfide isomerase or thioredoxin	-1.08	0.049

Table S2. MICs (mg/L) of murepavadin for indicated *P. aeruginosa* strains

Strains[#]	PA14	Δ*algU*	Δ*algU*/*algU*	*algD*::Tn
MIC	0.0625	0.0625	0.0625	0.0625

[#] | Data represent the results of three independent experiments.

Table S3. MICs (mg/L) of β-lactam antibiotics for PA14 strain

Antibiotics[#]	CB[a]	MEM[a]	CAZ[a]	CAZ/AVI[a,b]
MIC	75	0.5	2	2

[#] | Data represent the results of three independent experiments.

[a] | CB, carbenicillin; MEM, meropenem; CAZ, ceftazidime; AVI, avibactam.

[b] | Avibactam was fixed at 4 μg/mL.

Table S4. Clinical isolates used in this study

Strain[#]	Isolation sites	MIC (mg/L)			Carbapenemase
		Mur[a]	CAZ/AVI[a]	MEM	
CI-PA-17	Burn wound secretions	0.0625	4	>64	KPC
CI-PA-18	Burn wound secretions	0.03125	4	>64	KPC
CI-PA-19	Burn wound secretions	0.03125	4	>64	KPC
CI-PA-20	Burn wound secretions	0.03125	4	>64	KPC
CI-PA-21	Burn wound secretions	0.0625	8	>64	KPC
CI-PA-22	Burn wound secretions	0.0625	8	>64	KPC
CI-PA-36	Burn wound secretions	0.0625	4	>64	KPC
CI-PA-37	Burn wound secretions	0.03125	4	>64	KPC
CI-PA-38	Burn wound secretions	0.0625	4	>64	KPC
CI-PA-40	Burn wound secretions	0.03125	4	>64	KPC
CI-PA-41	Venous catheter tip	0.03125	4	>64	KPC
CI-PA-88	Sputum	0.03125	4	>64	KPC
CI-PA-90	Sputum	0.03125	4	>64	KPC
CI-PA-91	venous catheter tip	0.0625	4	>64	KPC

[#] | Data represent the results of three independent experiments.

[a] | Mur, murepavadin; CAZ, ceftazidime; AVI, avibactam; MEM, Meropenem.

Table S5. Killing effects on clinical isolates

	Mur[a] 0.5 mg/L		CAZ/AVI[a] 8/4 mg/L		Mur 0.5 mg/L+ CAZ/AVI 8/4 mg/L	
Strain	Survival	SD[*]	Survival	SD	Survival	SD
CI-PA-17	5.41E+01	±2.47E+00	4.97E-03	±4.75E-03	3.20E-05	±1.91E-05
CI-PA-18	1.56E+00	±2.55E-01	6.07E-02	±4.90E-02	2.32E-05	±1.57E-05
CI-PA-19	1.79E+00	±4.16E-01	6.71E-01	±3.09E-01	1.36E-05	±9.88E-06
CI-PA-20	5.45E+01	±4.17E+01	8.86E-01	±1.29E-01	4.24E-05	±1.44E-05
CI-PA-21	9.71E-03	±4.99E-03	9.75E-01	±1.93E-01	1.18E-05	±8.01E-06
CI-PA-22	3.36E-02	±9.04E-03	9.44E-04	±3.93E-04	7.41E-07	±3.21E-07
CI-PA-36	2.91E-04	±1.29E-05	5.45E-02	±6.43E-03	6.67E-06	±5.25E-06
CI-PA-37	1.31E+00	±9.62E-01	6.61E-01	±2.32E-01	1.95E-06	±1.52E-06
CI-PA-38	5.33E+01	±6.01E+01	1.15E+00	±6.19E-01	1.85E-04	±5.14E-05
CI-PA-40	4.89E+01	±5.46E+01	7.40E-01	±4.77E-02	1.17E-04	±1.00E-05
CI-PA-41	4.25E+01	±3.43E+01	7.97E+00	±2.69E+00	2.09E-05	±8.06E-06
CI-PA-88	3.77E+01	±1.75E+01	1.29E+00	±2.23E-01	4.47E-05	±1.12E-05
CI-PA-90	1.24E+01	±6.78E+00	3.73E-03	±4.42E-03	5.00E-05	±5.59E-05
CI-PA-91	2.25E-02	±1.16E-02	2.19E-02	±1.62E-02	1.53E-04	±5.43E-05

[*] | SD Standard deviation

[a] | Mur, murepavadin; CAZ, ceftazidime; AVI, avibactam.

Table S6. Bacterial strains, plasmids and primers used in this study

Strain	Description	Source
P. aeruginosa		
PA14	Wild type strain of *P. aeruginosa*	(1)
Δ*algU*	PA14 deleted of *algU*	This study
Δ*algU*/*algU*	Δ*algU* complementation with *algU* inserted on chromosome, Gm^r	This study
PA14*algD*::Tn	PA14 with *algD* disrupted by insertion of Tn; Gm^r	(1)
PA14/pUCP20NP -*lptF*-His	PA14 containing pUCP20NP-*lptF*-His; Cb^r	This study
PA14/pUCP24-*lpt F*	PA14 containing pUCP24-*lptF*; Gm^r	This study
CI-PA-17	Clinical isolate	This study
CI-PA-18	Clinical isolate	This study
CI-PA-19	Clinical isolate	This study
CI-PA-20	Clinical isolate	This study
CI-PA-21	Clinical isolate	This study
CI-PA-22	Clinical isolate	This study
CI-PA-36	Clinical isolate	This study
CI-PA-37	Clinical isolate	This study
CI-PA-38	Clinical isolate	This study
CI-PA-40	Clinical isolate	This study
CI-PA-41	Clinical isolate	This study
CI-PA-88	Clinical isolate	This study
CI-PA-90	Clinical isolate	This study
CI-PA-91	Clinical isolate	This study
E. coli		
DH5α	F-,φ80d*lacZ*ΔM15,Δ(*lacZYA-argF*)U169,*deoR*, *recA1*,*endA1*, *hsdR17*(r_k^-,m_k^+), *phoA*, *supE44*, λ^-, *thi-1*, *gyrA96*, *relA1*	TransGen
S17-1	*recA*, *pro*, *hsdR*, RP4-2-Tc::Mu-Km::Tn7	TransGen
Plasmids		
pEX18Tc	Gene replacement vector, TET^r, *oriT*$^+$, *sacB*$^+$	(2)
pEX18Tc-Δ*algU*	*algU* gene of PA14 deletion on pEX18Tc; Tc^r	This study
pUC18T-mini-Tn 7T-Gm- *algU*	pUC18T-mini-Tn7T-Gm with wild type *algU*; Gm^r	(2)
pUCP20	Broad-host-range cloning vector	(2)
pUCP20NP	Broad-host-range cloning vector without promoter	This study
pUCP24	Broad-host-range cloning vector, Gm^r	(2)
pUCP20NP-*lptF*-His	pUCP20NP harboring PA14 *lptF* cassette with a C-terminal His-tag	This study

pUCP24-*ampC*	*ampC* gene from PA14 cloned in pUCP24, Gm^r	This study
pUCP24-*lptF*	*lptF* gene from PA14 cloned in pUCP24, Gm^r	This study
pUCP24-*btuB*	*btuB* gene from PA14 cloned in pUCP24, Gm^r	This study

Primers	Sequence 5'-3'	Function
q*rpsL*F	CAAGCGCATGGTCGACAAGAG	RT-PCR
q*rpsL*R	ACCTTACGCAGTGCCGAGTTC	RT-PCR
q*mucA* F	GCAGATGGCGCAACAGGG	RT-PCR
q*mucA* R	CGGCGGATTGTTGCACGT	RT-PCR
q*algU* F	CACCAGACCATCCAGCAGT	RT-PCR
q*algU* R	TCTTCGTAACTCAAACCTTCG	RT-PCR
q*algD* F	CTGCCTGCCCAAGGATGT	RT-PCR
q*algD* R	TGTCGTGGCTGGTGATGAG	RT-PCR
q*lptF* F	GACAAGTCCGACCTGAAGC	RT-PCR
q*lptF* R	TAGCCCTCTACGATCACCTG	RT-PCR
algU-Up-F	CCGGAATTCGGGCACGGAAGGTATCGACT	*algU* deletion
algU-Up-R	GGCTTCTCGCAACAAAGGCTGCCGTTCAAC CAGTTGCTGATCC	*algU* deletion
algU-Down-F	GGATCAGCAACTGGTTGAACCAGCCTTTGT TGCGAGAAGCC	*algU* deletion
algU-Down-R	CGCGGATCCGACCAGGCGCAGGTCGTAC	*algU* deletion
Com-*algU*-F	CGGGATCCGCCTTCCTCGTGGTTGTGCT	*algU* cloning for chromosome insertion
Com-*algU*-R	CCCAAGCTTCCGCTTCGTTATCCATCACAG	*algU* cloning for chromosome insertion
ampC F	GCTCTAGATCCCGGGGCGGTTTCTCATGCA GCC	*ampC* cloning
ampC R	CCCAAGCTTCGCTACGCTCCGTCGCCCTCGC GAG	*ampC* cloning
lptF-His F	CGGGATCCGCGGTATTCGCCGACATG	*lptF*-His cloning on pUCP20NP
lptF-His R	CCCAAGCTTTCAATGGTGATGGTGATGATG GCCGCTGACGGAGGAGC	*lptF*-His cloning on pUCP20NP
lptF F	CGGGATCCGATTCCGCCCTTCTTTCGTC	*lptF* cloning on pUCP24
lptF R	CCCAAGCTTTCAGCCGCTGACGGAGGAGC	*lptF* cloning on pUCP24
btuB F	CGGGATCC ACGGAGCAGCGCAGAG	*btuB* cloning on pUCP24
btuB R	CCCAAGCTTTCACAGGGCCTGGTAGCGC	*btuB* cloning on pUCP24
KPC F	GGGGTACCACCTAGCTCCACCTTCAAAC	PCR for *KPC*

		detection
KPC R	CGGGATCCTTCAGAGCCTTACTGCCCG	PCR for *KPC* detection

Supplemental Methods.

Strain and plasmid construction

Construction of the in-frame deletion mutant in *P. aeruginosa* was performed as mentioned previously (3). To construct the *algU* deletion mutant, a 1039 bp and a 988 bp fragments upstream and downstream of the *algU* coding region were amplified by PCR using PA14 chromosomal DNA as the template with primers *algU*-Up-F, *algU*-Up-R and *algU*-Down-F, *algU*-Down-R (Table S6). The PCR products of the *algU* upstream and downstream fragments were cloned into the *EcoR*I-*Hind*III site of the plasmid pEX18Tc, respectively. The obtained plasmid was transferred into an *E. coli* conjugation donor strain S17-1 by electroporation, and then transferred to PA14 by conjugation. The PA14 single-crossover mutants (strains with plasmid integrated into the chromosome) were selected on LB plates with 50 μg/mL tetracycline and 25 μg/mL kanamycin (to kill the S17-1 donor strain). Single-crossover mutants were grown in LB overnight, and then selected for double-crossover mutants on plates containing 7.5% sucrose. Construction of the correct deletion mutants were screened by PCR with primers *algU*-Up-F and *algU*-Down-R.

For the complementation of *algU* through chromosomal insertion, a fragment containing the promoter region and *algU* open reading frame was amplified by PCR with primers Com-*algU*-F, Com-*algU*-R (Table S6). The PCR product was cloned into the *BamH*I-*Hind*III sites of the plasmid pUC18T-mini-Tn7T-Gm (2). The

resulting plasmid was transferred into the Δ*algU* mutant by conjugation. Selection of strains with the fragment insertion was screened as previously described (2).

References

1. Liberati NT, Urbach JM, Miyata S, Lee DG, Drenkard E, Wu G, Villanueva J, Wei T, Ausubel FM. 2006. An ordered, nonredundant library of *Pseudomonas aeruginosa* strain PA14 transposon insertion mutants. *Proc Natl Acad Sci U S A* 103:2833-2838.
2. Choi KH, Schweizer HP. 2006. mini-Tn7 insertion in bacteria with single attTn7 sites: example *Pseudomonas aeruginosa*. *Nat Protoc* 1:153-161.
3. Hoang TT, Karkhoff-Schweizer RR, Kutchma AJ, Schweizer HP. 1998. A broad-host-range Flp-FRT recombination system for site-specific excision of chromosomally-located DNA sequences: application for isolation of unmarked *Pseudomonas aeruginosa* mutants. *Gene* 212:77-86.
